# Investigation of critical factors influencing the underestimation of hearing loss predicted by the ISO 1999 predicting model

**DOI:** 10.1186/s12889-023-17138-w

**Published:** 2023-11-13

**Authors:** Fei Li, Hong-wei Xie, Shi-biao Su, Hua Zou, Li-Fang ZHou, Qiu-Liang Xu, Fang Wei, Mei-bian Zhang

**Affiliations:** 1 Zhejiang Provincial Center for Disease Control and Prevention, Occupational Health and Radiation Protection Institute, Hangzhou, Zhejiang China; 2 Guangdong Province Hospital for Occupational Disease Prevention and Treatment, Guangzhou, China; 3 Chinese Center for Disease Control and Prevention, National Institute of Occupational Health and Poisoning Control, Beijing, China

**Keywords:** Kurtosis, Noise-induced hearing loss, Permanent threshold shift, Predicting model, Underestimation

## Abstract

**Objective:**

To analyze factors influencing the underestimation of noise-induced permanent threshold shift (NIPTS) among manufacturing workers, providing baseline data for revising noise exposure standard.

**Design:**

A cross-sectional study was designed with 2702 noise-exposed workers from 35 enterprises from 10 industries. Personal noise exposure level(*L*_*Aeq,8h*_) and noise kurtosis level were determined by a noise dosimeter. Questionnaires and hearing loss tests were performed for each subject. The predicted NIPTS was calculated using the ISO 1999:2013 model for each participant, and the actual measured NIPTS was corrected for age and sex. The factors influencing the underestimation of NIPTS were investigated.

**Results:**

The predicted NIPTS at each test frequency (0.5, 1, 2, 3, 4, or 6kHz) and mean NIPTS at 2, 3, 4, and 6kHz (NIPTS_2346_) using the ISO 1999:2013 model were significantly lower than their corresponding measured NIPTS, respectively (*P* < 0.001). The ISO model significantly underestimated the NIPTS_2346_ by 12.36 dB HL. The multiple linear regression analysis showed that noise exposure level, exposure duration, age, and kurtosis could affect the degree of underestimation of NIPTS_2346_. The generalized additive model (GAM) with (penalized) spline components showed nonlinear relationships between critical factors (age, exposure duration, noise level, and kurtosis) and the underestimated NIPTS_2346_.The underestimated NIPTS_2346_ decreased with an increase in exposure duration (especially over ten years). There was no apparent trend in the underestimated NIPTS_2346_ with age. The underestimated NIPTS_2346_ decreased with the increased noise level [especially > 90 dB(A)]. The underestimated NIPTS_2346_ increased with an increase in noise kurtosis after adjusting for the noise exposure level and exposure duration and ultimately exhibiting a linear regression relationship.

**Conclusions:**

The ISO 1999 predicting model significantly underestimated the noise-induced hearing loss among manufacturing workers. The degree of underestimation became more significant at the noise exposure condition of fewer than ten years, less than 90 dB(A), and higher kurtosis levels. It is necessary to apply kurtosis to adjust the underestimation of hearing loss and consider the applying condition of noise energy metrics when using the ISO predicting model.

## Introduction

The World Health Organization (WHO) estimates that 10% of the global population is exposed to noise pollution, and 5.3% suffer from noise-induced hearing loss [1]. Occupational noise exposure is one of the leading causes of adult hearing loss [2]. Regarding its temporal structure, industrial noise can be divided into steady-state, continuous (Gaussian), and (non-Gaussian) complex noise. A complex noise environment is described as Gaussian background noise punctuated by a series of high-level transient noises, resulting in a non-Gaussian distribution. These transients may be brief, high-level noise bursts, impulses, or impacts with varying interpeak intervals, peak levels, and peak durations. Manufacturing workers are often exposed to complex noise environments such as electric wrenches,hand sanding, polishing and nail gunning et al. [3]. Workplace noise exposure is mainly complex noise with impulse or impact properties (also known as non-Gaussian or unsteady noise). In contrast, steady-state or Gaussian noise exposure is relatively rare [4].

Hearing risk assessment and measurement for noise exposure in existing standards (e.g., ISO 1999:2013 [5]) are based on the equal energy hypothesis (EEH) [3, 4]. However, there has yet to be a consensus on using simple energy metrics to predict noise's effects on hearing. Previous studies have shown that the EEH is usually considered appropriate for steady-state noise, but it is not adequate for complex noise [4, 6–9]. Therefore, to fully evaluate the impact of noise on hearing, the temporal structure of noise must be considered. Several studies using animal models have shown that exposure to complex noise produces more hearing damage than continuous Gaussian noise at equivalent noise energy [10–15]. These animal results which was supported by human data from industrial settings [6, 8, 16–19], have demonstrated that ISO 1999 model is not sufficient to evaluate the hearing loss caused by complex (non-Gaussian) noise exposure, and probably underestimate the risk of noise-induced hearing loss [2, 4, 8, 9, 19–22].

One metric effectively reflecting temporal variables (e.g., interpeak intervals, peak levels, and peak durations) is needed. Kurtosis [*β(t)*] statistic proposed by Erdreich [23] is defined as the ratio of the fourth-order central moment to the squared second-order moment of the amplitude distribution, which can be used to quantify the temporal structure of complex noise. Animal models [12–15] and human studies [3, 18–20] have shown that kurtosis is a critical metric to assess the hearing loss associated with complex noise, and can be an additional metric for noise energy metrics. Therefore, using the kurtosis metric to distinguish the risk of hearing damage at the same equivalent noise level (*Leq*) has attracted more scholars' attention.

Several studies have shown that the prediction model of ISO 1999 may underestimate the hearing damage caused by steady-state noise and unsteady noise exposure. For example, Davis et al. reported that the ISO 1999 predicting model might underestimate the hearing damage caused by exposure to steady and unsteady noise, especially for high-kurtosis levels of unsteady noise. Henderson et al. found that the noise-induced permanent threshold shift (NIPTS) of railway workers were underestimated by 9 dB at 2 and 4 kHz frequencies when using the ISO 1999 predicting model [24]. Leensen et al. also found that construction workers exposed to noise had more severe hearing loss than the predicted level; when the exposure duration is less than ten years, the predicted NIPTS_346_ was underestimated by 10 dB HL [21]. Zhang et al. conducted a human study on workers exposed to noise in various industries. They found that the ISO 1999 predicting model underestimated the NIPTS_346_ by 13.6 dB on average [19]. Wang et al. also found that the ISO 1999 predicting model underestimated the risk of NIHL in the machinery manufacturing industry and the NIPTS_2346_ by 10.7 dB on average [25].

The above underestimation by the ISO 1999 prediction model is associated with the noise's temporal structure (expressed by kurtosis). However, a research gap must be narrowed, i.e., the underestimated degree and its changing trend must be clarified. The role of other factors, such as age and energy metrics (noise level and exposure duration) influencing the underestimation, should be considered together. It is necessary to conduct further research on the relationship between of the degree of underestimation and critical factors, including kurtosis. This study applied a cross-sectional survey with a relatively large sample size of workers from multiple industries. By comparing the measured NIPTS with the predicted NIPTS by the ISO predicting model, the degree of underestimation of NIPTS was evaluated; critical factors (such as age, noise level, exposure duration, and kurtosis) influencing the underestimated NIPTS among manufacturing workers were investigated, which attempted to provide a basis for the revision of noise exposure measurement and assessment standard.

## Materials and methods

### Study design

A cross-sectional study was used for this study. The primary study elements included the selection of workplaces, recruitment of subjects, collection and analysis of noise waveforms, audiometry testing, prediction of NIPTS, and statistical analysis of critical factors influencing the estimation of NIPTS.

### Workplace selection

Workplace selection for this study was based on criteria assuring both Gaussian and non-Gaussian noise exposure existed in the workplace and a sufficient subject pool. Each workplace included must have the following:

1. A workforce that was stable over three years;
2. Production processes and machinery that were stable for at least five years;
3. Work areas with high levels of Gaussian and complex noise exposure longer than one year.

Before the data collection, a hygienist interviewed the administrators of the investigated factories to verify that the working environment remained constant. The research team members conducted field investigation to preliminarily evaluate the noise types and levels and understand the distribution of noise sources, enterprise products, production processes, the number of workers exposed to the noise, and measures taken to reduce the noise level. These selection criteria were designed to facilitate accurate cumulative noise exposure assessments for each worker. A total of 35 factories from ten specific manufacturing industries were investigated, e.g., textile, paper making, furniture, automobile, metal product, general equipment manufacturing, steel making, and so on.

### Subject recruitment

A total of 2702 workers exposed to high-level noise from 35 factories were recruited in Zhejiang, China, from 2010 to 2018. All candidate subjects were required to complete a noise exposure and health questionnaire, followed by a face-to-face interview. Individual information on general personal information (e.g., age, sex, smoking, and alcohol use) and information on occupational history (e.g., factory, workshop, type of job, noise exposure duration, and HPD use) and general health conditions (e.g., history of ear disease, use of ototoxic drugs, blood pressure, diabetes, and genetic diseases) was collected for each participant.

The subject's hearing loss is directly attributed to the measured industrial noise exposures and is unrelated to other medical problems. The ideal subject is one whose high-level noise exposure originates from the undertaking job and who has been employed at that same job for their entire employment history. Participant inclusion criteria were as follows:

1. Continuously working within the same job category and work site for their entire employment period and noise exposure level ≥ 80 dB(A), continuous noise exposure for more than one year;
2. No history of exposure to ototoxic medications, head wounds, or ear diseases;
3. No history of military service or shooting activities;
4. No or minimal use of hearing protection (determined from the noise exposure questionnaire and interview);
5. No history of diabetes;
6. No co-exposure to ototoxic organic solvents and heavy metals

Most participants still did not often use HPD despite the implementation of hearing conservation programs on a wide scale in China starting in 2012. HPDs, usually earplugs, were assessed through field observations by the researchers and in the questionnaire and reported to be low and infrequent. These participants would have a dose–effect relationship between noise level and NIPTS. We expected this effect to occur primarily in participants exposed to at least 95 dB(A) noise. For those participants who have never used HPDs, the research team members recommended using appropriate HPDs after data collection. The participants were asked to sign informed consent forms, ensuring they understood the study's purpose, procedures, and contents. The study protocol was approved by the ethics committee of the Zhejiang Center for Disease Control and Prevention, China (Zhejiang CDC, approval reference number: ZJCDC-T-043-R).

### Noise measurement and analyses

A digital recorder with a ¼-inch pre-polarized condenser microphone (ASV5910-R, Hangzhou Aihua Instruments Co., China) was used to obtain each participant's shift-long noise recordings. The instrument has a broad measurement range [40–141 dB(A)] and frequency response (20 Hz-20 kHz). The instrument microphone is relatively sensitive (2.24 mV/Pa) [3, 8, 26–28]. The continuous working time and the noise sampling rate of the recorder are 23 h and 48 kHz, respectively. The full-shift (usually 8 h) noise waveform was recorded for each participant. Noise measurement and evaluation methods were based on our previous studies on non-steady noise exposure and NIHL [3, 27, 28].

Before recording, a hygienist determined that each participant was exposed to the typical noise in the workplace, and trained the participants on how to wear the recorder properly [3, 26–28]. The recorder was clipped on each worker’s shoulder. The recorded noise signal was analyzed using a program designed by the MATLAB software to obtain the noise exposure metrics, e.g., A-weighted SPL (i.e., 8-h continuous equivalent, *L*_*Aeq,8 h*_), peak SPL, and kurtosis[*β(t)*] [8, 26–28]. Each kurtosis value of a consecutive 60-s time window was computed over the measurement duration [26–28]. The mean of all measured kurtosis values was calculated and used as the kurtosis of noise exposure (*β*_*N*_) [17, 29, 30].

### Audiometric test and analysis

Each participant underwent a general physical and otologic examination. Otoscopy was carried out initially to ensure participants had no external ear abnormalities. Air conduction pure tone HTLs were tested at 0.5, 1, 2, 3, 4, 6, and 8kHz in each ear by a certified audiologist according to the Chinese national standard (GB/T 16296.1–2018, originated from the ISO 8253–1:2010) at least 16h after the last occupational noise exposure. The audiometric test was performed in an audiometric booth [background noise < 30dB(A)] on a mobile medical examination vehicle using an audiometer (Madsen OB40, Denmark) with an air conduction headphone (Sennheiser HDA 300). The tests were conducted manually. Each participant's hearing data was recorded on a separate audiogram form, and all the data were entered into a computer after the daily test was completed. Before the test, the audiometer and headphones were calibrated for hearing thresholds by the Zhejiang Institute of Metrology of China, according to the Chinese national standard (GB/T 4854.1–2004, originated from the ISO 389–1:1998). The hearing thresholds were measured following the ascending procedure in 5 dB steps according to ISO 8253–1:2010.

### Analysis of Predicted Median NIPTS and Measured NIPTS

Measured HTLs at each frequency were adjusted by subtracting the age- and sex-specific HTL according to Table B.3 of ISO 1999 [3, 5, 8]. The database B.3 demonstrates selected values of the statistical distribution of hearing threshold levels in decibels of an unscreened population from the United States, where subjects with occupational noise exposure have been excluded. The average median noise-induced permanent threshold shift (NIPTS) of both ears at 2, 3, 4, and 6kHz frequencies (NIPTS_2346_) was calculated using the Formula 2 [3, 5, 26–28].

The ISO 1999 median NIPTS prediction for each subject was calculated using Fomula 1:

1 $$NIPTS=\left\{\frac{\left[u+vlg\left(\frac{t}{{t}_{0}}\right)\right]{\left({L}_{EX,8h-}{L}_{0}\right)}^{2},10\le t\le 40}{\frac{1g\left(t+1\right)}{1g\left(11\right)}\left[{u+vlg\left(\frac{10}{{t}_{0}}\right)}\right]\left({L}_{EX,8h-}{L}_{0}\right)^{2},t<10}\right.$$

*L*_*EX,8 h*_ is the noise exposure level normalized to a nominal 8h working day[dB(A)]; *t* is the exposure duration (years), *t*_*0*_ = 1; *L*_*0*_ is the sound pressure level, defined as a function of frequency in Table 1 of ISO 1999, below which the effect on hearing is negligible; *u* and *v* are given as a function of frequency in Table I of ISO 1999 [3, 5, 8, 26–28].

**Table 1:** General information and noise-exposure characteristics among participants

Industry	Number of Factories	n	Gender(n)		Age (year)	Duration (year)	L_EX,8 h_ [dB(A)]	Mean Kurtosis (*β*_*N*_)
			Male	Female				
Textile and Chemical fiber	3	258	130	128	34.34 ± 8.26	6.91 ± 4.44	93.56 ± 5.58	12.44 ± 13.33
Furniture	6	328	284	44	35.08 ± 9.83	5.03 ± 4.67	88.66 ± 3.81	120.52 ± 78.36
Automobile	7	960	782	178	35.00 ± 7.82	10.12 ± 7.90	89.12 ± 4.57	24.29 ± 27.67
Metal products	3	136	81	55	39.99 ± 9.42	11.67 ± 8.32	91.65 ± 5.63	20.91 ± 31.78
Electronic equipment	3	215	97	118	33.01 ± 7.93	6.53 ± 5.40	87.00 ± 3.84	33.25 ± 29.43
Paper	2	91	60	31	46.87 ± 9.74	10.33 ± 6.50	89.31 ± 3.71	10.92 ± 10.30
General equipment	5	387	289	98	38.91 ± 8.45	13.34 ± 6.22	90.52 ± 5.43	31.57 ± 27.93
Pipe parts	2	69	65	4	31.26 ± 9.22	5.00 ± 4.17	88.30 ± 4.42	31.38 ± 15.98
steel	3	179	179	0	38.60 ± 7.00	13.44 ± 7.86	93.76 ± 5.42	36.69 ± 47.33
Child carriage production	1	79	42	37	39.87 ± 8.48	4.50 ± 3.91	93.57 ± 3.85	20.34 ± 23.67
Summary	35	2702	2009	693	36.28 ± 8.88	9.38 ± 7.27	90.07 ± 5.13	36.86 ± 49.48

The NIPTS_2346_ was calculates using the Formula 2:

2 $${NIPTS}_{2346}=\frac{NIPTS_2+NIPTS_3+NIPTS_4+NIPTS_6}4$$

The frequency range of 2, 3, 4, and 6kHz was used in this study because NIHL occurs early in this frequency range [6, 10]. Considering that the participants’ noise exposure was relatively stable during their working life, the estimates of hearing loss were likely attributable to the actual exposure to industrial noise. Comparing the predicted NIPTS for each exposure condition to the actual NIPTS under the same exposure condition was feasible. This study plans to use four hearing loss-related metrics (i.e., noise exposure level, exposure duration, age, and kurtosis level) to evaluate the level of NIPTS underestimation [3, 6, 10].

### Cumulative noise energy assessment

The *CNE*, a composite noise exposure index [23], was used to quantify the noise exposure for each subject. The CNE is defined as:

3 $$CNE=101g\left[\frac{1}{{T}_{ref}}\sum_{i=1}^{n}\left({T}_{i}\times {10}^{{L}_{Ae{q}^{,8hi/10}}}\right)\right]$$

where* L*_*Aeq,8 h*_ is the equivalent continuous A-weighted noise exposure level in decibels normalized to an 8-h working day;occurring over the time interval T_i_ in years; with a total of n different noise level exposure periods (i.e.,years spent working in different noise tasks/environments); and T_ref_ = 1 year. For all subjects in this study, *n* = 1 (as all workers were restricted to being exposed to only one occupational noise environment) and Eq. (1) can be reduced to:

4 $$CNE={L}_{Aeq,8h}+101gT$$

To evaluate the underestimation of the ISO 1999 predicting model, participants were classified into different groups according to their noise exposure level, exposure duration, age, and kurtosis level.

Noise level group:(1)*L*_EX,8 h_ < 85 dB(A),(2)85 ≤ *L*_EX,8 h_ < 88 dB(A), (3)88 ≤ *L*_EX,8 h_ < 91 dB(A),(4) 91 ≤ *L*_EX,8 h_ < 94 dB(A),(5)94 ≤ *L*_EX,8 h_ < 97 dB(A),(6) L_EX,8 h_ ≥ 97 dB(A).

Exposure duration: (1)Duration < 5 years,(2) 5 ≤ Duration < 10 years, (3) 10 ≤ Duration < 15 years,(4) 15 ≤ Duration < 20 years,(5)Duration ≥ 20 years.

Age group:(1)age < 25 years,(2) 25 ≤ age < 30 years, (3) 30 ≤ age < 35 years,(4) 35 ≤ age < 40 years,(5)40 ≤ age < 45 years,(6)age ≥ 45 years.

Kurtosis level:(1) *β*_*N*_ < 10,(2)10 ≤ *β*_*N*_ < 30, (3) 30 ≤ *β*_*N*_ < 50,(4) 50 ≤ *β*_*N*_ < 100,(5) *β*_*N*_ ≥ 100.

This approach allowed us to understand better:(1)the influence factors of the degree of underestimation and their applicable conditions and (2) the relative contribution of the temporal features (kurtosis) on the level of underestimation.

### Statistical analyses

Multiple linear regression was used to analyze the influence of each factor on the degree of underestimation. One-way analysis of variance (ANOVA) was used to analyze the overall difference of underestimation between different groups. The two analyses were performed using IBM SPSS Statistics (version 22). In addition, the generalized additive model (GAM), with (penalized) spline components, which was performed using R software(version 4.2.2), was used to explore factors' variation trends and capture the unknown functional relationship. The models are compared using Akaike's information criterion (AIC) and the proportion of explained variability. The minimum AIC is chosen as the more reasonable model; simultaneously, the number of nodes is selected according to the minimum AIC principle. Estimated spline functions are presented along with their point wise 95% confidence intervals. All the tests are evaluated at a significance level of 0.01.

## Results

### General characteristics of noise exposure among participants

Table 1 lists the general characteristics of the participants. Workers are mainly aged between 22 and 48 (87.7%). The mean age of these workers was 36.29 years. The exposure duration ranged from 1 to 30 years; the mean exposure duration was 9.4 years, and 55.9% of the workers had an exposure duration between 5 and 20 years. The noise exposure level was between 80 and 110dB(A), and the average noise exposure level was 90.07dB(A). The percentage of male workers was 74.4%. The kurtosis noise level ranged from 3 to 300, mainly concentrated between 10 and 200 (72.3%), with an average value of 36.86.

### Comparisons between the predicted NIPTS and measured NIPTS across different hearing threshold test frequencies

The T-test in Table 2 shows that the predicted NIPTS at each frequency (0.5 kHz, 1 kHz, 2 kHz, 3 kHz, 4 kHz, or 6 kHz) were significantly less than the measured NIPTS(*P* < 0.001). The ISO 1999 prediction model significantly underestimated the NIPTS_2346_ by 12.27 dB HL(95% CI: 11.83–12.70, *P* < 0.001). One-way ANOVA showed no overall difference among the frequency groups (*P* > 0.01).

**Table 2:** Differences between the measured NIPTS and the predicted NIPTS at different frequencies

Frequency group	Measured NIPTS (dB HL)	Predicted NIPTS (dB HL)	Underestimated NIPTS (dB HL)		
			Mean	Standard Error	95% CI
500 Hz	13.71 ± 7.41	0.47 ± 1.74	13.23^a^	0.15	12.95–13.53
1000 Hz	14.94 ± 6.92	0.98 ± 2.32	13.97^a^	0.14	13.69–14.24
2000 Hz	15.05 ± 8.64	3.01 ± 3.87	12.04^a^	0.18	11.68–12.39
3000 Hz	19.61 ± 12.89	8.46 ± 7.82	11.15^a^	0.26	10.63–11.66
4000 Hz	23.13 ± 14.93	10.75 ± 8.80	12.38^a^	0.30	11.79–12.97
6000 Hz	20.76 ± 15.01	7.26 ± 6.60	13.50^a^	0.30	12.92–14.08
NIPTS_2346_	19.64 ± 10.63	7.37 ± 6.73	12.27^a^	0.22	11.83–12.70

^a^*P* < 0.001, the measured NIPTS vs the predicted NIPTS

### Regression analysis of critical factors influencing the NIPTS underestimation

In order to explore critical factors influencing the degree of underestimation, multiple linear regression was used to analyze the relationship between the mean underestimation value of NIPTS_2346_ and several factors such as noise level, exposure duration, kurtosis, age, and gender. Table 3 shows that* L*_*EX,8h*_ had the highest contribution to the degree of underestimation, and the exposure duration, kurtosis, and age were important factors influencing the degree of underestimation.

**Table 3:** Regression analysis of factors influencing the degree of underestimation

Variable	Estimate(*B*)	*B*(95% CI)	*P* value
Gender	-0.604	-1.523 ~ 0.314	0.198
Age (year)	0.113	0.060 ~ 0.166	< 0.01
Exposure duration (year)	-0.341	-0.406 ~ -0.275	< 0.01
*L*_*EX,8 h*_ [dB(A)]	-0.678	-0.755 ~ -0.600	< 0.01
Kurtosis	0.029	0.021 ~ 0.037	< 0.01

### The role of exposure duration in the degree of NITPS underestimation

A trend test (Table 4) showed that the degree of NIPTS _2346_ underestimated decreased with an increase in noise exposure duration (*F* = 134.771, *P* < 0.001). One-way ANOVA showed that the underestimation value of NIPTS_2346_ in the 10–15, 15–20, or ≥ 20 years group was significantly less than the < 5- or 5–10 years group (*P* < 0.001); the underestimation value of NIPTS_2346_ in the 15–20, or ≥ 20 years group was significantly less than the 5–10 or 10–15 years group (*P* < 0.001); the underestimation value in the ≥ 20 years group was significantly less than the 15–20 years group (*P* < 0.001).

**Table 4:** The effect of exposure duration on the degree of NITPS_2346_ underestimation

Exposure duration (year)	n	Measured NIPTS_2346_ (dB HL)	Predicted NIPTS_2346_ (dB HL)	Underestimated NITPS_2346_ (dB HL)		
				Mean	Standard Error	95% CI
< 5	930	18.33 ± 8.67	3.90 ± 3.37	14.43	0.29	13.85–15.00
5–10	627	19.93 ± 10.28	6.60 ± 4.96	13.33	0.42	14.49–14.16
10–15	507	20.97 ± 11.60	8.76 ± 6.17	12.22^a^	0.55	11.14–13.29
15–20	323	20.51 ± 11.73	10.61 ± 7.99	9.90^abc^	0.71	8.51–11.30
≥ 20	315	19.89 ± 13.17	13.61 ± 9.57	6.28^abcd^	0.78	4.73–7.83

^a ^*P* < 0.001, compared with the < 5 years group

^b ^*P* < 0.001, compared with the 5–10 years group

^c ^*P* < 0.001, compared with the 10–15 years group

^d ^*P* < 0.001, compared with the 15–20 years group

**Fig. 1:**
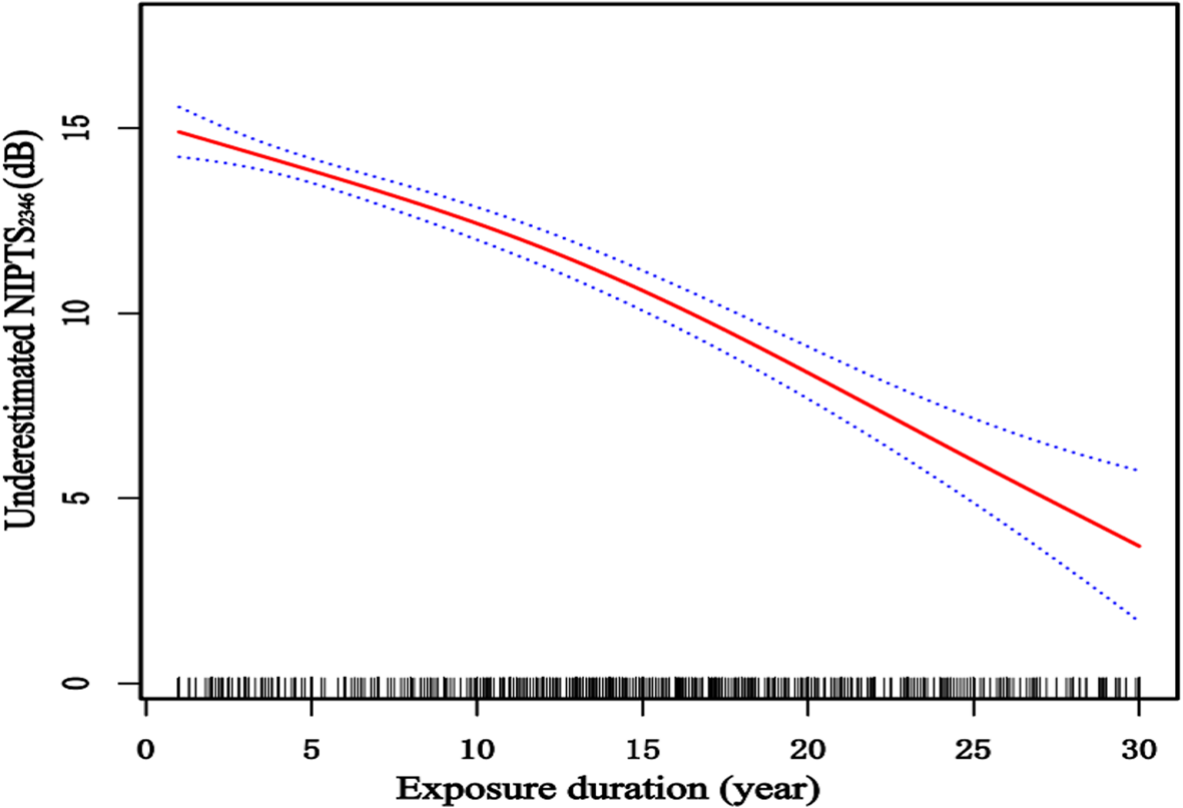
The relationship between exposure duration and the level of NIPTS underestimation

In order to visually observe the relationship between the degree of NIPTS underestimation and exposure duration, the generalized additive model, GAM,with (penalized) spline components to explore their changing trends. The minimum AIC is chosen as the more reasonable model; simultaneously, the number of nodes is selected according to the minimum AIC principle. Figure 1 shows no simple linear relationship between the underestimation and exposure duration. When the exposure duration was less than ten years, the level of underestimation decreased with the increase in the exposure duration is not apparent. When the exposure duration was more than ten years, the level of underestimation significantly decreased with the increase in the exposure duration.

### The effect of age on the degree of NIPTS_2346_ underestimation

One-way ANOVA showed a significant overall difference between the age groups (*F* = 16.618, *P* < 0.001). The Bonferroni adjustment was applied for multiple comparisons. Table 5 shows a relatively higher underestimation for the 25–30 years group than other groups (*P* < 0.001).

**Table 5:** Comparison of the mean NIPTS_2346_ underestimation among different age groups

Age(year)	n	Actual measured NIPTS_2346_(dB HL)	predicted NIPTS_2346_(dB HL)	NIPTS_2346_ Underestimation(dB HL)		
				Mean	Standard Error	95% CI
< 25	265	16.75 ± 6.30	3.69 ± 3.38	13.06	0.42	12.23–13.89
25–30	429	20.46 ± 8.51	4.66 ± 4.84	15.79^a^	0.43	14.95–16.64
30–35	468	18.07 ± 9.93	7.00 ± 5.92	11.08^b^	0.49	10.11–12.04
35–40	560	20.11 ± 11.09	8.22 ± 7.03	11.89^b^	0.50	10.91–12.86
40–45	470	19.13 ± 11.01	9.78 ± 8.20	9.35^abc^	0.55	8.26–10.44
≥ 45	510	21.84 ± 13.01	8.75 ± 7.03	13.09^bd^	0.62	11.87–14.31

^a ^*P* < 0.001, compared with the < 25 years group

^b ^*P* < 0.001, compared with the 25–30 years group

^c ^*P* < 0.001, compared with the 35–40 years group

^d ^*P* < 0.001, compared with the 35–40 years group

**Fig. 2:**
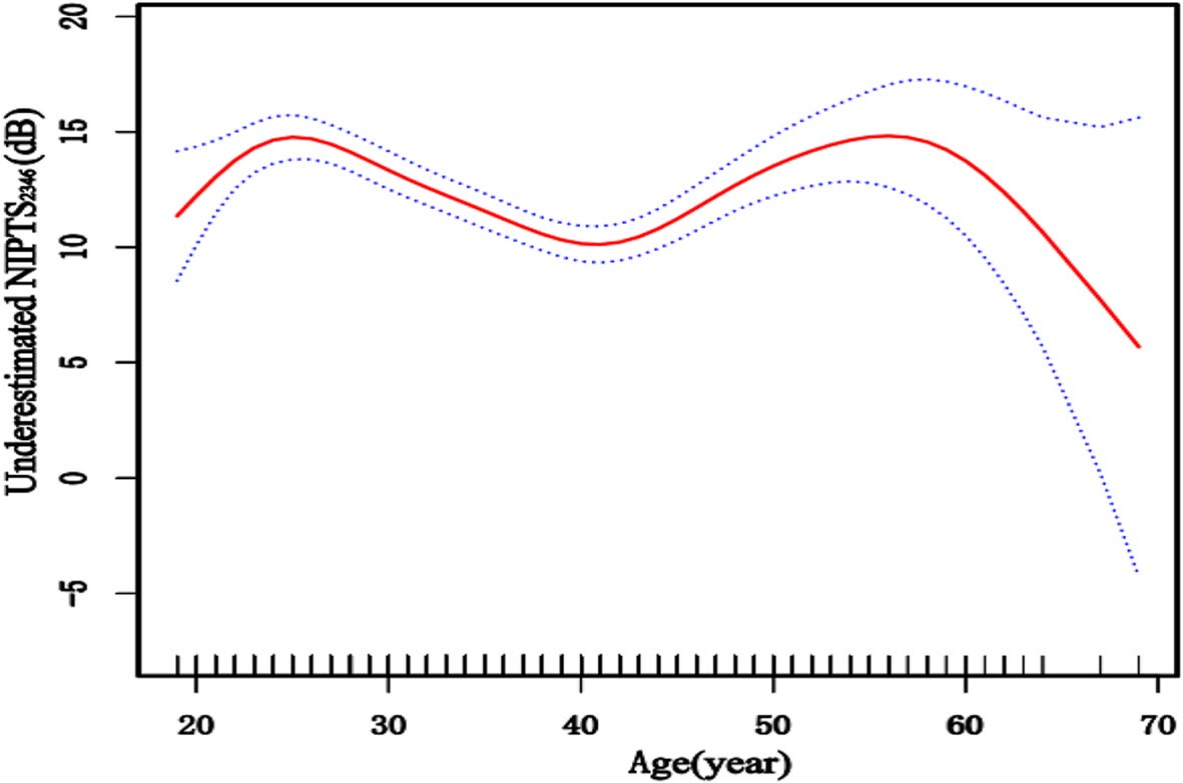
The relationship between age and the level of underestimated NIPTS

To visually confirm the relationship between two variables, the generalized additive model, GAM,with (penalized) spline components, was used to explore their variation trends. Figure 2 shows that there was not a simple linear relationship or apparent trend between the underestimated NIPTS_2346_ and age.

### The effect of noise level on the degree of NIPTS_2346_ underestimation

One-way ANOVA in Table 6 showed a significant overall difference between the groups of noise level (*F* = 73.479, *P* < 0.001). The Bonferroni adjustment, which was applied for multiple comparisons, showed that the underestimated NIPTS_2346_ in the 91–94 dB(A) and ≥ 94dB(A) groups were significantly lower than other noise-level groups (*P* < 0.001). According to the trend test, the underestimated NIPTS_2346_ decreased with the noise level increase (*F* = 293.330, *P* < 0.001).

**Table 6:** Comparison of the underestimated NIPTS_2346_ among different noise level groups

*L*_*EX,8 h*_ [dB(A)]	*n*	actual measured NIPTS_2346_(dB)	Predicted NIPTS_2346_(dB)	NIPTS_2346_ Underestimation(dB HL)		
				Mean	Standard Error	95% CI
< 85	455	16.54 ± 8.59	1.39 ± 0.74	15.15	0.40	14.36–15.95
85–88	538	18.36 ± 9.79	3.34 ± 1.37	15.01	0.42	14.19–15.84
88–91	620	19.61 ± 10.19	5.48 ± 2.31	14.12	0.42	13.31–14.94
91–94	514	21.26 ± 11.73	8.89 ± 3.27	12.37^abc^	0.52	11.34–13.39
94–97	288	20.87 ± 11.13	12.69 ± 4.77	8.18^abcd^	0.68	6.84–9.51
≥ 97	287	22.87 ± 11.87	20.42 ± 8.51	2.45^abcde^	0.78	0.91–3.99

^a ^*P* < 0.001, compared with the < 85dB(A) group

^b ^*P* < 0.001, compared with the 85-88dB(A) group

^c ^*P* < 0.001, compared with the 88–91 years group

^d ^*P* < 0.001, compared with the 91–94 years group

^e ^*P* < 0.001, compared with the 94–97 years group

**Fig3:**
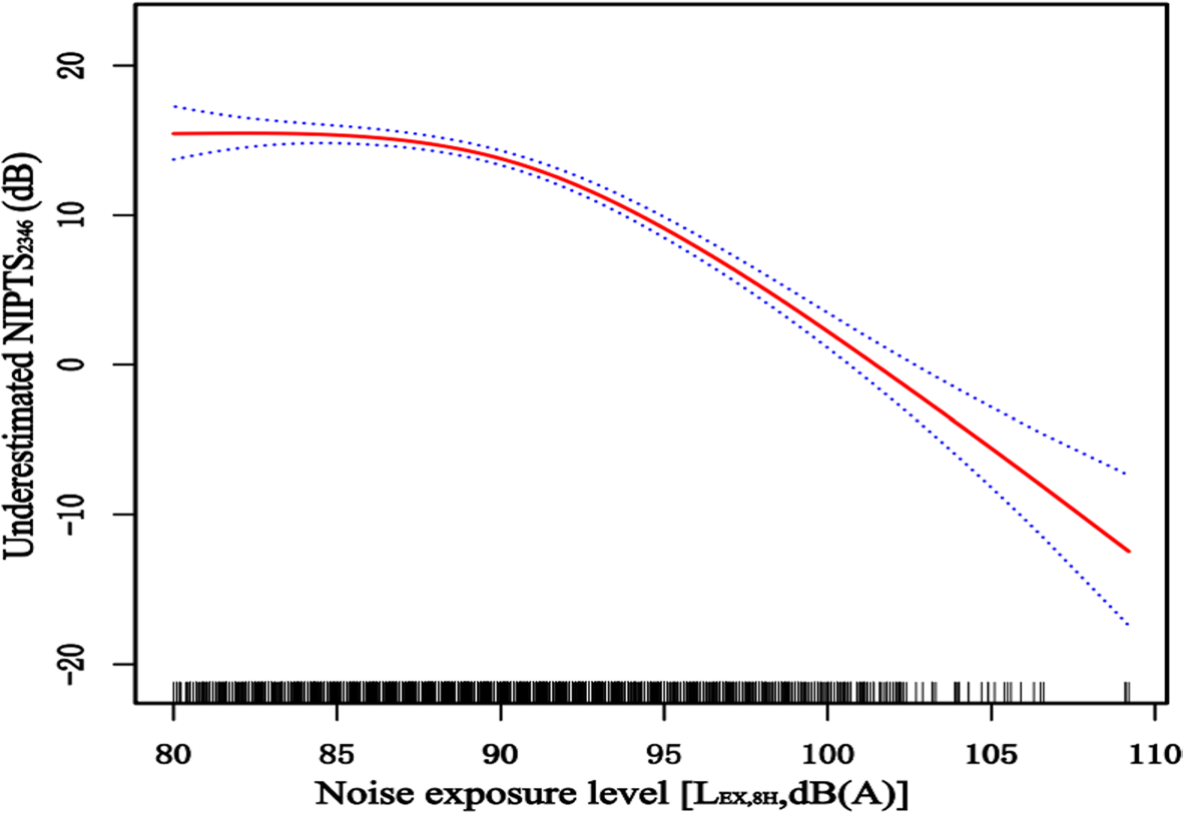
The relationship between noise exposure level and the level of underestimation

According to the above results, the level of underestimation is related to the noise exposure level. General additive models demonstrated that when the noise exposure level is less than 91dB(A), the level of underestimation does not change significantly with the increase of the noise exposure. However, when the noise exposure level is higher than 91dB(A), the level of underestimation will decrease significantly with the noise exposure level. The trend result is shown in Fig. 3.

### The effect of kurtosis level on the degree of NIPTS_2346_ underestimation

A trend test (Table 7) showed that the degree of NIPTS_2346_ underestimated decreased with an increase in noise exposure duration (*F* = 76.615, *P* < 0.001). One-way ANOVA showed a statistically significant overall difference between the groups of kurtosis level (*F* = 21.289, *P* < 0.001). Bonferroni adjustment was applied for multiple comparisons. Table 7 showed that the underestimation value of NIPTS_2346_ in the *β(N)* 10–30,50–100, or ≥ 100 group was significantly more than the < 10 group (*P* < 0.001); the underestimation value of NIPTS_2346_ in the 50–100, ≥ 100 group were significantly more than the 10–30 or 30–50 group (*P* < 0.001).

**Table 7:** Comparisons of the underestimated NIPTS_2346_ among different noise kurtosis groups

*β(N)*	n	Measured NIPTS_2346_(dB)	Predicted NIPTS_2346_(dB)	Underestimated NIPTS_2346_ (dB HL)		
				Mean	Standard Error	95% CI
< 10	668	19.08 ± 9.99	9.13 ± 7.58	9.94	0.42	9.12–10.76
10–30	1139	19.03 ± 10.16	6.87 ± 6.51	12.17^a^	0.33	11.53–12.81
30–50	398	19.46 ± 11.27	7.52 ± 6.85	11.94	0.60	10.76–13.12
50–100	263	21.35 ± 11.88	6.57 ± 5.77	14.79^abc^	0.74	13.33–16.25
≥ 100	234	22.56 ± 11.50	5.45 ± 4.82	17.11^abc^	0.77	15.59–18.64

^a ^*P* < 0.001, compared with the < 10 group

^b ^*P* < 0.001, compared with the 10–30 group

^c ^*P* < 0.001, compared with the 30–50 years group

**Fig. 4:**
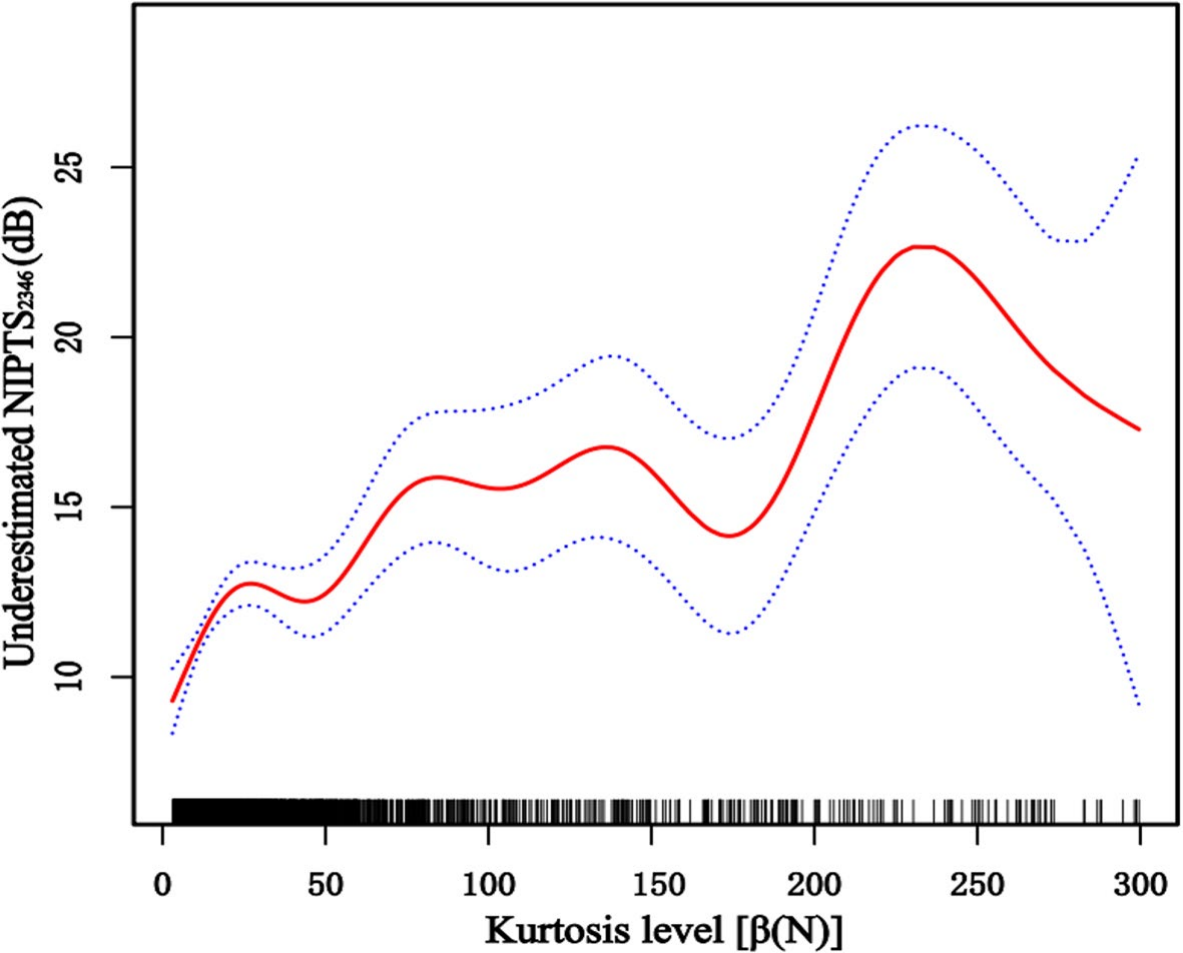
The relationship between kurtosis level and underestimated NIPTS

**Fig. 5:**
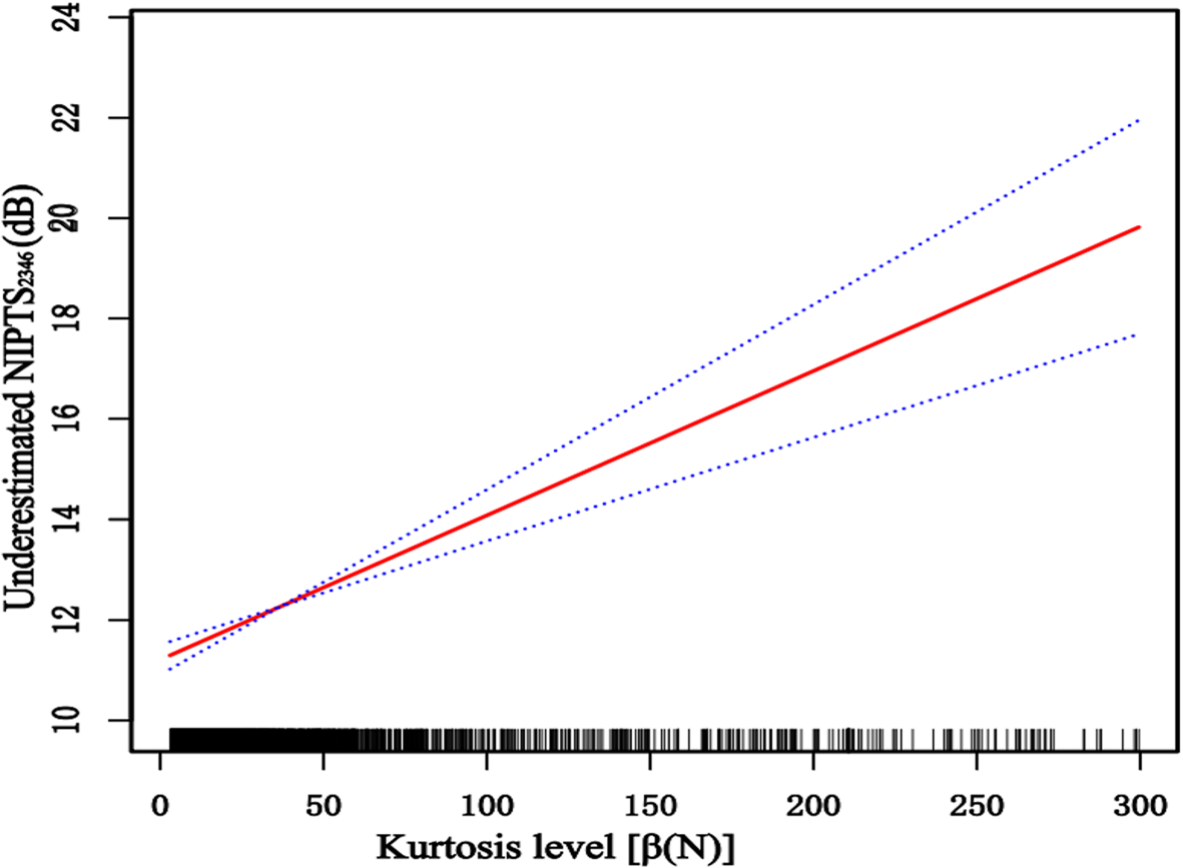
The relationship between kurtosis level and underestimated NIPTS after adjusting noise exposure duration and noise level

According to the above results, the level of underestimation was related to the exposure noise kurtosis. General additive models demonstrated that when the kurtosis level is lower than 300, Fig. 4 shows that the degree of underestimation tended to increase with noise kurtosis. The correlation between kurtosis level and underestimation was further analyzed after adjusting the exposure duration and noise level. Figure 5 shows that the underestimated NIPTS_2346_ was linearly correlated with noise kurtosis level; the degree of underestimation increased with an increase in the kurtosis level.

### Linear regression between underestimated NIPTS _2346_ and kurtosis level

**Fig. 6:**
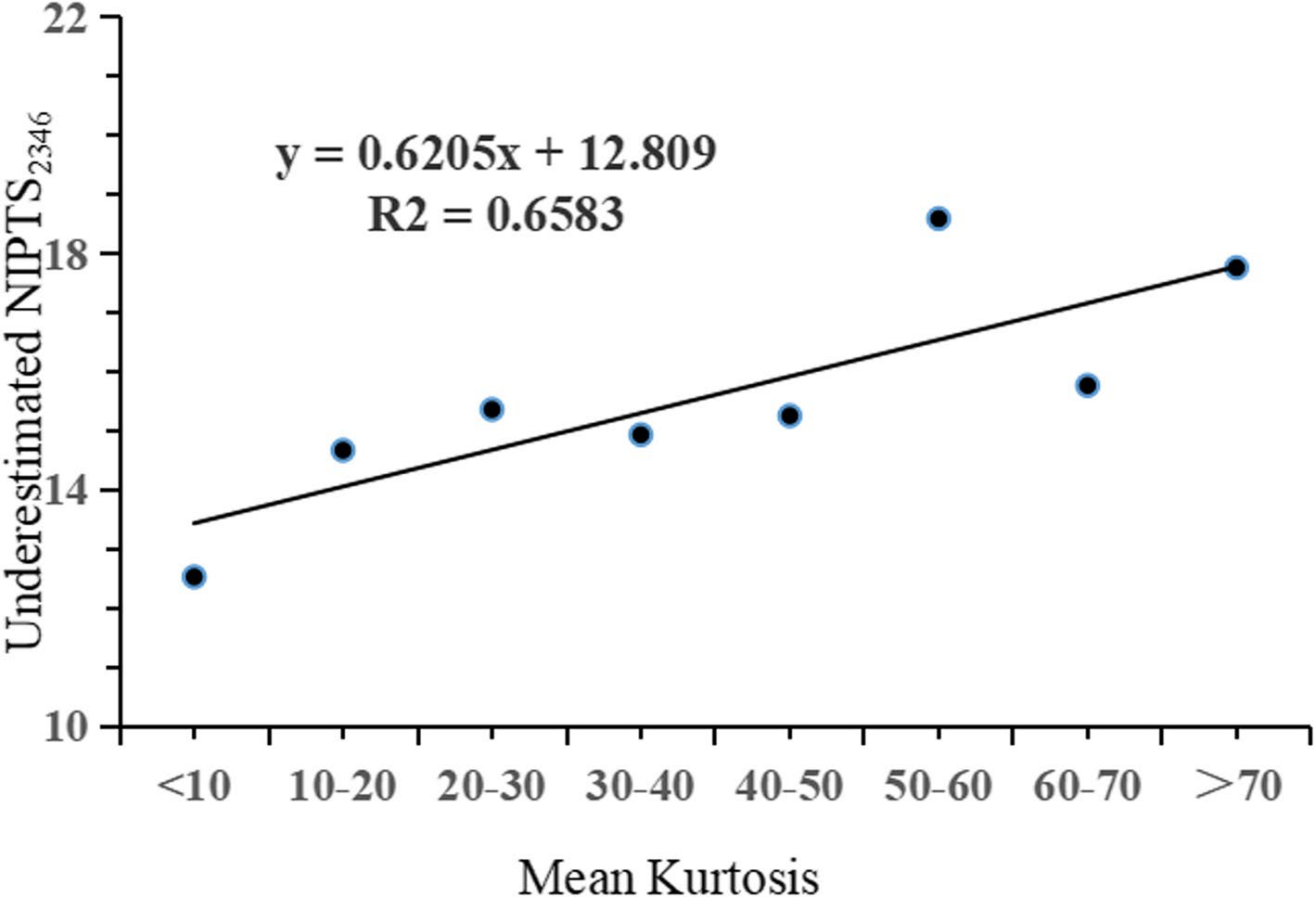
A linear regression relationship between underestimated NIPTS_2346_ and kurtosis

Based on the above research results, a noise energy metric (*CNE*), which can comprehensively reflect the exposure level and duration, was used to quantify the noise exposure for each subject. The *CNE* index was used to study further the quantitative relationship between the underestimation level and noise kurtosis. Participants with *CNE* < 100 were taken as sub-subjects and divided into eight groups according to their kurtosis level: < 10, 10–20, 20–30, 30–40, 40–50, 50–60, 60–70, and > 70. Figure 6 shows a linear regression equation between the underestimated NIPTS_2346_ and kurtosis (y = 0.6205χ + 12.809, *R*^2^ = 0.6583).

## Discussion

### ISO 1999 predictive model underestimated the median NIPTS

The results from the present study indicated that the test frequencies included 0.5,1,2, 3, 4, and 6kHz; compared with the actual measured NIPTS, the predicted NIPTS at each hearing threshold test frequency underestimated the noise-induced hearing loss by an average of 12.36 dB. The results are consistent with several studies which reported that the ISO model underestimated the amount of NIPTS. For example, Zhang et al.compared the predicted average NIPTS_2346_ by the ISO 1999 model with the actual measured NIPTS corrected for age and sex among 2,133 screened workers from 34 industries in China; the authors reported that the ISO 1999 predictions significantly underestimated the NIPTS by 7.5 dB on average in Gaussian-noise exposed workers and by 13.6 dB on average in workers exposed to non-Gaussian noise with high kurtosis [3]. Davis et al. reported that the ISO 1999 predictive model significantly underestimated (up to ~ 10–15 dB at 2 or 6 kHz) the median NIPTS in most noise-exposed groups [8]. Hearing thresholds of railway workers were reported to exceed ISO 1999 predictions by 9 dB over 2 and 4 kHz frequencies [24]. Leensen et al. analyzed hearing thresholds in 29,644 construction workers in the Dutch industry, they found that the ISO 1999 model was inconsistent with the population data during the first ten years of exposure, i.e., the pure-tone average at 3, 4, and 6 kHz was about 10 dB poorer than that predicted by ISO 1999 [21]. Barry Lempert evaluated the ISO 1999 model and found lower estimates of the risk of noise-induced hearing impairment by using ISO 1999 [22].

These study's results further illustrate the inaccuracy of using ISO 1999 to evaluate noise-induced hearing loss. A more accurate model needs to consider the role of kurtosis instead of only energy metrics. The possible reasons are: (1) ISO 1999 documents are based on data for NIHL, and high level noise exposures were almost derived from steady or quasi-steady industrial noises and collected several decades ago; (2) All noise exposures are quantified by a single metric (energy metric) without considering the temporal structure of the noise; (3) The energy metric single is insufficient to characterize non-Gaussian (complex) noise exposure.

### Investigation of critical factors influencing the underestimation

The possible influencing factors of the underestimation were studied, and it was found that the exposure duration, age, noise level, and kurtosis level may affect the degree of underestimation of the noise-induced hearing loss. Regarding exposure duration's potential influence of underestimated NIPTS_2346_, participants were classified into six exposure groups. Compared with the actual measured NIPTS_2346_, the predicted NIPTS_2346_ in each duration group significantly underestimated the noise-induced hearing loss. The amount of underestimation was significant in the < 10 years group compared with other ≥ 10 years groups. When the exposure duration was less than ten years, the amount of underestimation decreased as the exposure duration increased, but it was insignificant. In contrast, the trend chart showed that the degree of underestimation decreased significantly with an increase in exposure duration when the exposure duration was greater than ten years. These results may be related to the development process of noise-induced hearing loss. NIHL develops most rapidly in the first 10 years and then slows with additional exposure to noise [21]. Once NIHL has manifested, it worsens slightly with continued noise exposure [31]. Hence in the first 10 years, the hearing loss increases rapidly with exposure duration, the underestimation by the ISO 1999 prediction model might be relatively apparent. When the exposure duration is more than ten years, the noise-induced hearing loss slows down gradually, and the NIPTS underestimation might be relatively unapparent. However, the degree of underestimation gradually decreased with the increased exposure duration when the exposure duration was more than ten years.

In this study, participants were classified into six noise exposure groups. Compared with the actual measured NIPTS_2346_, the ISO 1999 predicted NIPTS_2346_ of each noise level group significantly underestimated the noise-induced hearing loss. When the noise level was 80-91dB(A), the degree of underestimation was significantly higher than other noise level groups. The trend chart (Fig. 3) illustrates that the underestimation degree gradually decreased with an increase in the noise level; Especially when the noise level was more than 94 dB(A), the degree of underestimation decreased significantly. Previous studies showed a dose–response relationship between exposure to noise and hearing loss, that was, higher exposure levels and longer exposure duration cause more severe hearing impairment [21, 32, 33], this relationship is mathematically described in ISO 1999. Leensen et al. conducted a retrospective study on 29,644 construction workers, and it showed that up to exposure levels of 91 dB(A), construction workers exhibited greater hearing loss than predicted; when workers were exposed to low noise levels, workers' hearing loss was much greater than predicted, whereas at high noise levels, hearing loss was less [21]. These results agree this study's finding that hearing loss was much greater than predicted at low noise exposure levels, whereas, at high noise levels, hearing loss was less. Therefore, the amount of underestimation decreased significantly, when the noise level was more than 94 dB(A). Another possible reason is that some participants who are exposed to higher noise level of more than 90 dB(A) sporadically wear earplugs and other personal protective equipment [21], the measured NIPTS may be relatively slightly lower than the actual possible hearing loss; as a result, the amount of underestimation decreases at high noise exposure levels. These findings remind us of the need to consider the applicable range of noise exposure duration and noise exposure levels when using the ISO 1999 model to predict NIPTS in the future.

Previous studies have shown that kurtosis may effectively discriminate the risk of hearing loss and sensory cell loss for noise exposures with equal energy but different temporal characteristics; NIHL increased as the kurtosis increased [3, 12, 14, 34]. The use of an energy metric (*Leq*) in combination with the kurtosis of the amplitude distribution of a noise environment can be used to more accurately estimate the hazards to hearing loss from the diversity of complex noise environments found in the industry [3, 34]. Davis et al. found that the ISO 1999 predictive model was not as accurate in estimating the median NIPTS incurred from high-level kurtosis industrial noise as it assessed low-level kurtosis industrial noise; the extent to which the ISO model underestimated the median NIPTS was more significant for the higher kurtosis level than the lower kurtosis level among the noise conditions across the test frequencies [34]. Zhang et al. found that the extent of the underestimation of NIPTS by the ISO 1999 increased with an increase in noise kurtosis value. For a fixed range of noise exposure level and duration, the actual measured NIPTS increased as the noise kurtosis increased; The noise with kurtosis of more than 75 produced the highest NIPTS [3]. The results of this study are consistent with those of previous animal studies and population epidemiological studies. In this study, participants were classified into five exposure groups to evaluate the effect of kurtosis level on the underestimation of NIPTS_2346_. Compared with the actual measured NIPTS_2346_, the ISO 1999 predicted NIPTS_2346_ of each kurtosis level group significantly underestimated the noise-induced hearing loss. The trend chart demonstrated that the underestimation gradually increased with the increased kurtosis level when the kurtosis value was less than 300. After adjusting noise exposure level and exposure duration, it was found that the degree of underestimation increased with the increase of kurtosis level, and the two variables showed a linear regression trend. It is further indicated that kurtosis can be used as an essential noise parameter to evaluate hearing loss caused by noise. These findings indicate kurtosis can be used to establish new noise measurement and assessment standards.

In this study, we only analyzed NIPTS data with noise exposure of 80–110 dB (A). However, the lower applicability limit of 75 dB (A) is also included in the NIPTS calculation method in the ISO 1999 model. In addition, in this study, the number of research subjects with more than 30 years of exposure duration and kurtosis levels of more than 300 in the database needs to be increased. In the future, we need further to collect the primary data on exposure noise levels of 75–80 dB (A), exposure duration of more than 30 years, and kurtosis levels of more than 300 from various industrial enterprises, for establishing a more extensive sample database and conducting in-depth research.

The observed NIPTS by subtracting the median of the expected HTLA distributions (from the B.3 database of the ISO 1999) from the observed PTS. While the predicted NIPTS were approximated by the median of the ISO1999 NIPTS distributions. The distributions of the observed PTS (consisting of NIPTS and PTA) are asymmetric in the different exposure-duration subgroups with different skewness values. In addition, the distributions of the expected NIPTS are narrower than those of the PTS. Therefore, the method of determining the observed NIPTS may lead to an underestimation, or at least part of it, what part of the underestimation may be due to the approximation of the predicted NIPTS with the medians of the calculated distributions.

## Conclusions

Based on the above findings, the ISO 1999 model significantly underestimated noise-induced hearing loss. Especially when the exposure duration was less than ten years or the noise exposure level was less than 94 dB(A), the degree of underestimation was most significant. The NIPTS_2346_ underestimation increased with an increase in kurtosis level, ranging from 9.81dB in the kurtosis < 10 groups to 17.69 dB in the kurtosis ≥ 100 groups. In addition, noise exposure duration, exposure level, and age were all associated with the NIPTS underestimation. When complex noises are prevalent in the workplace, applying the ISO 1999 model may require consideration of the application condition of noise levels and exposure duration. Kurtosis can be an essential parameter to evaluate hearing loss in the future to establish a new and more accurate method to predict the risk of hearing loss associated with complex noise.

## Data Availability

The datasets used and/or analysed during the current study available from the corresponding author on reasonable request.
